# *Ex vivo* localization of wireless implantable microdevice using high-resolution 3D imaging techniques

**DOI:** 10.3389/fbioe.2026.1830115

**Published:** 2026-06-02

**Authors:** Teresa Giannattasio, Michela Fratini, Francesco Brun, Luca Brombal, Manuela Montanaro, Valeria Palumbo, Antonio Canichella, João F. Ribeiro, Syed Bilal Nizami, Flavia Franceschini, Marco Micali, Eugenia Guida, Susanna Dolci, Luca Berdondini, Manuel Scimeca, Alessandro Mauriello, Allegra Conti, Nicola Toschi

**Affiliations:** 1 Department of Biomedicine and Prevention, Tor Vergata University, Rome, Italy; 2 Institute of Nanotechnology, CNR, Rome, Italy; 3 NeuroImmagini Lab, Santa Lucia Foundation, Rome, Italy; 4 Department of Engineering and Architecture, University of Trieste, Trieste, Italy; 5 Division of Trieste, Istituto Nazionale di Fisica Nucleare (INFN), Trieste, Italy; 6 Department of Physics, University of Trieste, Trieste, Italy; 7 Microtechnology for Neuroelectronics Unit, Fondazione Istituto Italiano di Tecnologia (IIT), Genova, Italy; 8 A.A. Martinos Center for Biomedical Imaging and Harvard Medical School, Boston, MA, United States

**Keywords:** 3D imaging, brain imaging, implantable devices, neurotechnology, phase-contrast tomography

## Abstract

The CROSSBRAIN EU project aims to address the heterogeneous nature of brain pathologies by developing wireless implantable microbots (µBots, planned dimensions 100 × 100 × 100 μm^3^) for highly localized neuromodulation. These devices are designed to precisely modulate brain activity with minimal invasiveness, enabling targeted resolution of specific spatiotemporal events, capabilities not currently achieved by existing neuromodulation technologies. A crucial step involves visualizing and ensuring the optimal placement of the µBots in the brain tissue, to study their functionality after implantation. In this preliminary *ex vivo* study, we used non-functional µBot silicon (Si) dummies matching the lateral dimensions of the intended µBots, with reduced thickness (100 × 100 × 50 μm^3^) to simplify fabrication and handling. Due to the intrinsic MRI incompatibility of the µBot platform, encompassing both the dummies used in this study and the future functional devices under development, and the limitations of standard histological approaches in reliably identifying and preserving the implant site during processing, we developed an integrated imaging workflow combining 2D and 3D techniques. While standard histological methods and tissue clearing presented substantial limitations in preserving the position of the dummies within the brain tissue, combining histological techniques with 3D X-ray tomography provided a robust strategy. In particular, synchrotron radiation-based X-ray Phase Contrast Tomography (XPCT), with its intrinsic high contrast and resolution, enabled detailed visualization of dummies within the surrounding vascular and cellular architecture. In contrast, conventional micro-Computed Tomography (micro-CT), although more widely accessible, enabled non-destructive guidance for targeted sectioning. Importantly, and in line with the scope of a Brief Research Report, this study presents a preliminary but technically robust investigation conducted within the CROSSBRAIN project, aimed at identifying and establishing an optimized imaging strategy for the visualization of implanted µBots in brain tissue. This methodological framework is intended as an initial step toward future *in vivo* studies, in which the validated imaging pipeline will be applied to track both dummy and functional devices and to enable subsequent evaluation of foreign body response under physiologically relevant conditions. This *ex vivo* workflow therefore provides the essential technical foundation for such future investigations and supports the clear positioning of this work as a feasibility and optimization study. This approach could be particularly valuable for new generations of implantable technologies incompatible with MRI and could support future development of personalized neuromodulation therapies by enabling precise device localization and structural tissue assessment.

## Introduction

Neurodegenerative and neuropsychiatric disorders are characterized by heterogeneous and dynamic alterations in specific neural circuits, often accompanied by abnormal electrographic activity such as ictal and interictal events (Naz and Siddique, 2020; Cano et al., 2021; Mukhtar, 2020; Chin and Scharfman, 2013; Zhang W. et al., 2023; Panagiotopoulou et al., 2022). In conditions such as Alzheimer’s and Parkinson’s disease, seizures can both arise from and exacerbate neurodegeneration (Cano et al., 2021; Mukhtar, 2020; DeTure and Dickson, 2019; Kouli et al., 2018), whereas in psychiatric disorders (e.g., depression, schizophrenia, bipolar disorder) abnormal activity in prefrontal–limbic circuits contributes to diverse clinical phenotypes (Chin and Scharfman, 2013; Robinson and Bergen, 2021). The marked inter-individual variability in the location, extent and temporal evolution of these pathological patterns complicates timely identification and targeted intervention.

Current neuromodulation approaches span non-invasive techniques such as transcranial magnetic stimulation (TMS), transcranial direct current stimulation (tDCS), and transcranial ultrasound stimulation (TUS), as well as invasive approaches such as deep brain stimulation (DBS) (Rossini et al., 2015; Woods et al., 2016; Darmani et al., 2022; Lozano et al., 2019). While clinically effective for selected indications, these approaches have limited ability to selectively target small neuronal populations embedded in deep structures with millisecond-scale temporal precision (Alfihed et al., 2024; Zhang S. et al., 2023). Non-invasive techniques are constrained by coarse spatial resolution and field spread, whereas DBS requires the implantation of macroscopic electrodes connected to subcutaneous pulse generators, with limited spatial sampling and fixed stimulation geometry (Lozano et al., 2019; Alfihed et al., 2024). The foreign body reaction (FBR) is a major concern for implanted devices, involving acute neurovascular damage, glial activation, and chronic inflammation, all of which affect neuronal viability, implant integration and device functionality over time (Kolaya and Firestein, 2021; Perna et al., 2023; Carnicer-Lombarte et al., 2021). Miniaturization of neuromodulation devices is therefore a promising strategy to improve spatial specificity, reduce tissue damage, and potentially mitigate FBR.

The CROSSBRAIN EU project (www.crossbrain.eu) aims at facilitating the development of personalized treatments for brain disorders by introducing new wireless, self-standing implantable micro-devices, here referred as microbots (µBots planned dimensions 100 × 100 × 100 μm^3^). These microscale devices are designed to provide highly accurate modulation of brain regions with minimal invasiveness and the ability to target specific spatiotemporal events (on the scale of a fraction of a millisecond with resolution compatible with stimulating individual neuronal cells). Their small size, wireless powering and data transmission technologies, and capability for deep brain implantation may allow their application through various neuromodulation techniques currently under study (such as mechanical, optogenetic, photo-thermal, and electro-chemical) with the goal of achieving stable long-term integration in brain tissue with a reduced FBR and improved interfacing stability compared to conventional electrodes. A crucial step to optimize their use involves visualizing and ensuring the optimal placement of the µbots post-implantation, as well as enabling future evaluation of tissue response at the implantation sites. The CROSSBRAIN project is intended for preclinical applications, with the goal of translating this technology for use in patients in the future.

A crucial step in optimizing the employment of µBots is the post-implantation visualization of device placement and tissue response. Due to their small size and material composition, µBots results incompatible with standard MRI. To avoid partial-volume effects, reliable visualization of a structure requires sampling by more than two voxels across its characteristic dimension (Marques and Norris, 2018; Tohka, 2014). Given that the µBot dimensions are approximately 100 μm, this implies a target voxel size of at most ∼50 µm to ensure adequate spatial sampling and detectability. Achieving such spatial resolution in vivo MRI remains technically challenging and is generally feasible only with ultra–high-field systems above 7 T (Boulant et al., 2024; Mulder et al., 2019), often in combination with advanced hardware solutions such as cryogenic RF coils or optimized phased-array coil designs to enhance signal-to-noise ratio at small voxel sizes (Dieringer et al., 2013). Non-MRI-compatible systems, including certain metal-based implants, highly specific molecular probes, or regions of dense brain tissue, further complicate conventional MRI-based imaging approaches due to susceptibility artifacts and contrast limitations (Winter et al., 2021). Alternative approaches such as CLARITY, micro-Computed Tomography (micro-CT), and synchrotron radiation-based X-ray Phase Contrast Tomography (XPCT) emerged as valuable tools for overcoming these obstacles and enabling 3D high-resolution visualization of the brain. CLARITY is a tissue-clearing technique that enables the visualization of cellular and molecular structures in three dimensions by rendering the brain optically transparent (Epp et al., 2015). Through hydrogel embedding and lipid removal, CLARITY preserves proteins, nucleic acids, and other biomolecules *in situ*, while allowing deep penetration of light for fluorescence imaging. This technique permits the examination of large brain volumes with minimal disruption to structural integrity. For systems that are MRI-incompatible, CLARITY in principle provides an avenue for high-resolution optical imaging of neural networks, synaptic connections, and cellular-level features in intact tissue volumes (Chung and Deisseroth, 2013); however, tissue clearing protocols, are known to induce morphological changes, such as tissue expansion or shrinkage, which can compromise the spatial integrity of rigid implants (Padgett, 2023), particularly at the microscale. Thanks to the development of micro-CT and its advanced variation, XPCT, it is now possible to obtain high-resolution 3D images of the entire brain with structural detail at the micrometer scale. Conventional micro-CT is particularly effective for visualizing dense materials, such as brain implants (Dalal et al., 2008), and is well suited for detecting features that are not compatible with MRI (Willemink et al., 2018). However, as reported in previous studies, its reliance on differences in X-ray attenuation limits its ability to distinguish soft tissues with similar densities, resulting in poor intrinsic contrast in brain tissue (Willemink et al., 2018). XPCT addresses this limitation by exploiting the phase shifts that X-rays undergo as they pass through different tissues, rather than relying solely on attenuation. Previous work has demonstrated that this imaging approach significantly improves soft tissue contrast compared to conventional micro-CT, enabling visualization of fine anatomical features, including neural and vasculature structures (Begani Provinciali et al., 2020; Bukreeva et al., 2017), and other components of the brain at microscopic resolution (Cedola et al., 2017; Massimi et al., 2019).

In this preliminary ex vivo-study, we focused on developing and evaluating an imaging pipeline using multiple approaches, including standard microscopy, CLARITY, micro-CT, and XPCT, to optimize post-implantation visualization. This study was not designed as a comparative evaluation of imaging modalities but rather aimed to explore various techniques to identify an effective workflow for locating µBots after implantation and analyzing tissue response around the implant’s side. Non-functional µBot silicon (Si) dummies (100 × 100 × 50 μm^3^) were used to mimic the material and footprint of planned µBot devices, but do not include functional components, multilayer architectures, or surface coatings. Therefore, this study does not address implantation mechanics, device stability, or biological response, but evaluates the feasibility of detecting and spatially localizing microscale implants *ex vivo*, allowing us to refine imaging parameters and establish procedures for future *in vivo* studies, including chronic evaluation of FBR and device integration.

## Methods

### μBot Si dummies fabrication process

The μBot silicon (Si) dummies (Lecomte et al., 2020) were produced at the Italian Institute of Technology (IIT, Genova, Italy), using the following process: a 200-nm-thick chromium (Cr) layer was deposited by Direct-Current (DC) sputtering onto a 50-μm-thick silicon wafer and patterned by photolithography to serve as a hard mask for the dry-etching process. A standard Bosch process was then employed to, etch the silicon and create the dummy μBots (see Supplementary Figure S1a), which were subsequently released in water after the wet etching of the Cr layer. The final size obtained for each dummy was 100 × 100 × 50 μm^3^. The quality of the shape, size and surface of the dummies was verified using Scanning Electron Microscopy (SEM) (Supplementary Figures S1b-d). The fabrication procedure for Si µBots dummies enables the production of hundreds of dummies per batch. However, for the purposes of this study, only those devices that were actually implanted in the specimens included in the analysis were considered (see details in Table 1 regarding the number).

**TABLE 1:** Overview of sample allocation, embedding and imaging method applied.

Number of brains	Embedding	X-clarity	XPCT	Micro-CT	Histology (H&E)	µBot Si dummies (number and site)
1	Paraffin	​	​	​	●	1 (right hemisphere)
1	Hydrogel	●	​	​	​	7 (right hemisphere)
1	Paraffin	​	●	●	●	2 (1 per hemisphere)
1	Paraffin	​	●	●	●	3 (2 right, 1 left hemisphere)

● Modality applied to the sample. Empty cell: modality not applied.

### *Ex vivo* tissue preparation

μBot Si dummies were implanted in *ex vivo* brain tissues from adult C57BL/6J mice (male of ∼30 g). Animals were anesthetized (ketamine 50 mg/kg + medetomidine 1 mg/kg) and perfused with an osmotic pump at a rate of 20 mL/min with 40 mL of 1X phosphate-buffered saline (PBS). Subsequently, tissues were fixed using 4% paraformaldehyde (PFA) solution in PBS. The brains were then removed and placed in a Petri dish. μBot Si dummies were resuspended in 3 µL of distilled water and gently inserted into the fixed brain tissue at approximately 1–2 mm depth at random cortical and subcortical sites using the pipette tip as a blunt inserter. Due to the manual nature of this procedure, precise control over the insertion site was not achievable, and the exact position of each dummy could not be predetermined beyond the hemisphere of implantation. The number of µBot Si dummies inserted varied across samples and is reported for each brain in Table 1. The brain tissues were subsequently incubated at 4 °C overnight in 50 mL of fresh 4% PFA solution. Different embedding methods were utilized based on the specific requirements of each analysis technique (see Table 1).

Four brains were used in this study (see Table 1). One brain was processed exclusively for conventional histology without prior 3D imaging to assess the limitations of unguided sectioning and one brain was dedicated to CLARITY-based tissue clearing. Two brains underwent a sequential multimodal workflow including XPCT, micro-CT, and subsequent CT-guided histological analysis. All implanted brains were included in the analysis; no selection of successful cases was performed.

All procedures were conducted in compliance with institutional and national regulations for animal research and were approved by the Italian Ministry of Health’s regulations (authorization no. 809/2024-PR).

### Imaging techniques

#### Light microscopy

Standard microscopy was performed using the Hematoxylin and Eosin (H&E) assay. Brain tissues were embedded in paraffin after implantation using a standard protocol as described in (Smart et al., 2023). Briefly, after fixation, the tissue was dehydrated using a series of alcohols (EtOH 70%, EtOH 80%, EtOH 100%) and then clarified in Xylene for at least 1 h. Incubation in paraffin at 60 °C for 60 min was followed by overnight incubation in fresh paraffin at 60 °C. After 24 h, the tissue was placed in biocassettes containing fresh paraffin solution and cooled at room temperature. Four micrometer thick sections obtained using a microtome were stained using a standard H&E assay protocol (Cardiff et al., 2014). Briefly, brain slices were deparaffinized in xylene, rehydrated in a graded ethanol series from 100% to 70%, rinsed in distilled water, and sequentially stained with hematoxylin and eosin. This assay results in blue-stained nuclei, while the cytoplasm and extracellular matrix exhibit varying shades of pink. Images were acquired under transmitted light using a ZEISS Axioscope seven microscope (Carl Zeiss Microscopy GmbH, Germany) equipped with a Colibri 3 LED illumination system, an Axiocam 305 color digital camera, and objectives with magnifications as indicated in the figure legends. Image acquisition was carried out using ZEISS ZEN 3.11 software.

#### X-CLARITY

Post-implanted brain tissue was clarified using the automated protocol provided by Logos Biosystems (X-CLARITY^TM^ Tissue Clearing System) as described in (Logos Biosystems, 2023). Briefly, after fixation, the whole brain was washed with 1X PBS to remove residual 4% PFA and incubated in a hydrogel solution (24 h, 4 °C). The next day, the hydrogel was polymerized using a vacuum chamber at −90 kPa and 37 °C for 3 h to allow tissue infiltration. Before the clearing step, the olfactory bulb and cerebellum were removed to reduce the volume being processed and thereby shorten the clearing time. The electrophoretic process was carried out with the following parameters: Voltage: 70 V, Constant Current: 1.2 A, Temperature: 37 °C, Rotation Speed: 50 rpm. During this process, electrophoresis was paused at several time points (0 h, 2 h, 4 h, 5.30 h) to determine the optimal time for dummies’ visualization. The protocol was completed after 5.30 h. The clarified brain was then sliced into 2-mm-thick sections using a razor blade. Images were acquired using a ZEISS Axioscope 7 microscope (Carl Zeiss Microscopy GmbH, Germany).

#### Micro-computed tomography (micro-CT)

X-ray micro-CT measurements were conducted at the PEPI laboratory of the Italian National Institute for Nuclear Physics (INFN, Trieste division) (Brombal et al., 2023). Whole µBot Si dummy-implanted-brain samples were embedded in paraffin before imaging. Brains were scanned using a micro-focus tube (Hamamatsu L10101) and a small-pixel photon-counting detector (Pixirad-PixieIII). The resulting tomographic datasets had an isotropic voxel size of 20 × 20 × 20 μm^3^. The tube voltage was 50 kVp, tube current = 100 μA, a 0.5 mm Al filter was used and 1440 projections having an exposure time of 2 s each were collected. Reconstructions were performed using the conventional FDK cone-beam algorithm with ring-artifact correction.

#### Synchrotron radiation-based X-ray phase contrast tomography (XPCT)

To evaluate 3D structural interface between tissue and the µBot Si dummies, *i.e.*, the spatial distribution of cells and vessels around the dummies, XPCT measurements in propagation mode were conducted at the SYRMEP beamline of the Elettra Synchrotron (Basovizza, Trieste, Italy) using a white beam with a mean energy of 22 keV, a pixel size of 3 × 3 μm^2^, and a propagation distance of 20 cm (Palermo et al., 2020). XPCT data preprocessing, phase retrieval, and tomographic reconstructions were performed using the open-source SYRMEP Tomo Project toolkit (Brun et al., 2017). Minimum and maximum intensity projection images of adjacent slice slabs were also analyzed to better visualize the tissue surrounding the µBots Si dummies. We acquired 3600 projections over 360° rotation following the so-called “half beam” acquisition with an exposure time of 0.1 s per projection. Paganin’s phase retrieval was applied, and parallel beam reconstruction was then performed. A detailed summary of sample allocation, tissue embedding and imaging modalities is reported in Table 1.

## Results

### Limitations of 2D histology due to device displacement and tissue tearing

**FIGURE 1:**
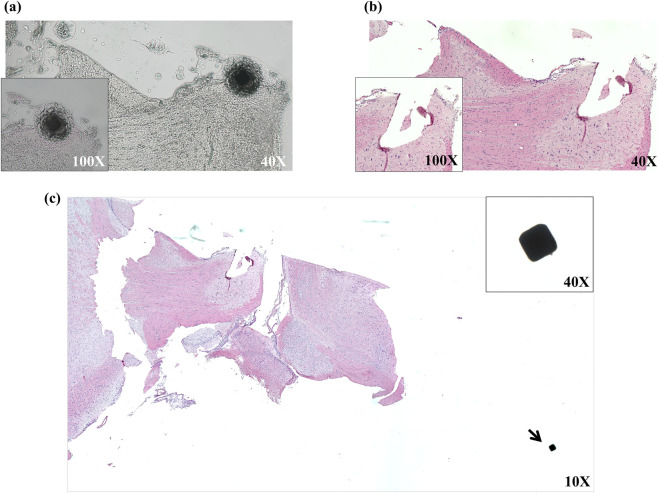
Standard microscopy analysis. Images before **(a)** and after **(b)** Hematoxylin and Eosin staining protocol. The top two panels show the implantation area with **(a)** and without **(b)** µBot Si dummy, highlighting the damage caused by the loss of the dummy during the staining process. **(c)** Tiled scan of the whole brain region section with µBot Si dummy completely dissociated from the tissue (black arrow); inset shows the relocated dummy on the glass slide. Objective magnifications are indicated in the figure.

Figure 1 illustrates the use of standard microscopy as an initial approach to identify µBot Si dummies within *ex vivo* brain tissue. Due to its higher density, the dummy can be distinguished from the surrounding soft tissue (Figure 1a). However, the H&E staining process revealed major limitations. Even when the dummy is correctly sectioned and initially retained within the tissue slice (Figure 1a), repeated washing steps during the staining process lead to its displacement from the original implantation site (Figure 1b). As shown in Figure 1c, the µBot Si dummy is found relocated onto the glass slide, completely dissociated from the tissue, resulting in loss of spatial correspondence with the surrounding tissue.

An additional limitation is associated with the microtomy process itself. Conventional histological sectioning is typically performed rapidly to obtain serial, consecutive sections; however, this approach becomes problematic in the presence of the µBot Si dummy. During sectioning, the rigid nature of the dummy induced severe mechanical disruption, causing the tissue slice to fragment into multiple pieces. This extensive tearing compromises both histological assessment and the preservation of recognizable tissue morphology. As shown in Supplementary Figure S2, panel Supplementary Figure S2a displays the hemisphere containing the implanted dummy, which appears highly fragmented as a result of sectioning, while Supplementary Figure S2b shows the contralateral hemisphere. This level of disruption prevents a continuous and reliable evaluation of the tissue surrounding the implant within a single section. This outcome was observed in 1/1 brain analyzed with standalone histology (100%), with complete loss of spatial correspondence between the dummy and the surrounding tissue, as reported in Table 2.

**TABLE 2:** Quantitative outcome summary across imaging modalities and histological approaches.

Outcome Measures	Standalone histology	CLARITY	Micro-CT	XPCT	CT-guided histology
Brains analyzed	1	1	2	2	2
Dummies implanted	1	7	5	5	5
Dummies successfully localized	0/1 (0%)	0/7 (0%)	5/5 (100%)	5/5 (100%)	5/5 (100%)
Dummy displacement or loss	1/1 (100%)	7/7 (100%)	0/5 (0%)	0/5 (0%)	0/5 (0%)
Details of displacement	Complete loss during staining	4/7 not retrieved; 3/7 retrieved outside implantation site (1 contralateral hemisphere, 2 outside implantation hole)	N/A	N/A	No displacement observed. Localized tissue disruption confined to implant region (reported separately in row below)
Histological sections with tissue tearing	Present: Section split in two-halves at implant site; dummy lost during staining; no spatial recovery possible	N/A	N/A	N/A	Present: ∼25 sections lost per dummy (∼100 µm ÷ 4 µm section thickness); intact sections successfully retrieved immediately before and after implant region
Inter-sample variability	N/A	High: Displacement extent varied across dummies	Low: All dummies localized consistently	Low: All dummies localized consistently	Low: Consistent retrieval of intact sections adjacent to implant

Together, these findings highlight a critical limitation of conventional 2D histology in preserving and accurately analyzing tissue architecture at the implantation site, particularly in the presence of rigid microscale devices.

### Limitations of X-CLARITY: tissue swelling and micro-device displacement

Given the limitations observed with conventional histological processing—namely tissue disruption during microtomy and loss of device positioning during staining—a three-dimensional tissue clearing approach was explored as an alternative to better preserve the implant–tissue interface. The outcomes of this method applied to brain tissue are shown in Supplementary Figure S3. At the end of the clarification protocol (after 5.30 h), the measured sample dimension approximately doubled its original volume (Supplementary Figure S3a). Microscopic examination of the cleared samples revealed significant displacement of the µBot Si dummies from their original implantation site. Of the seven dummies implanted, none was retrieved at the original implantation site. Four dummies were not retrieved at all, while three were found outside the original implantation site with variable displacement severity: two dummies were found outside the implantation hole at distances of approximately 650 μm and 867 µm from the center of the implantation site, respectively (Supplementary Figure S3b), while one dummy had migrated to the contralateral hemisphere at a distance of approximately 1.812 mm from the center of the implantation hole (Supplementary Figure S3c). This displacement is likely due to a combination of factors, including tissue swelling during lipid removal, hydrogel expansion, and mechanical forces induced by the reagent flow from the osmotic pump used in this specific protocol. While CLARITY-based techniques are widely adopted for volumetric imaging, morphometric alterations, such as tissue swelling or shrinkage, are commonly reported across different CLARITY protocols and are not specific to the X-CLARITY method applied in this study.

These changes, consistent with previously reported effects on micrometric morphological changes in the cleared tissue (Padgett, 2023), significantly compromised the spatial fidelity of the samples. In the present study, the observed displacement of the µBot Si dummies complicates the precise post-clearing identification of the original implantation dummies’ localization and, consequently, limits the ability to accurately assess local tissue responses in future *in vivo* experiments.

Consequently, these findings highlight a key limitation of tissue clearing for applications requiring micrometric spatial accuracy, particularly for evaluating implant localization and tissue–device interactions. As such, under the *ex vivo* conditions tested in this study, X-CLARITY did not preserve the original position of rigid silicon µBot Si dummies, precluding accurate assessment of implant localization and spatial relationships at the microscale, essential for our analyses.

### XPCT imaging for precise localization and tissue observation of µBot Si dummies

**FIGURE 2:**
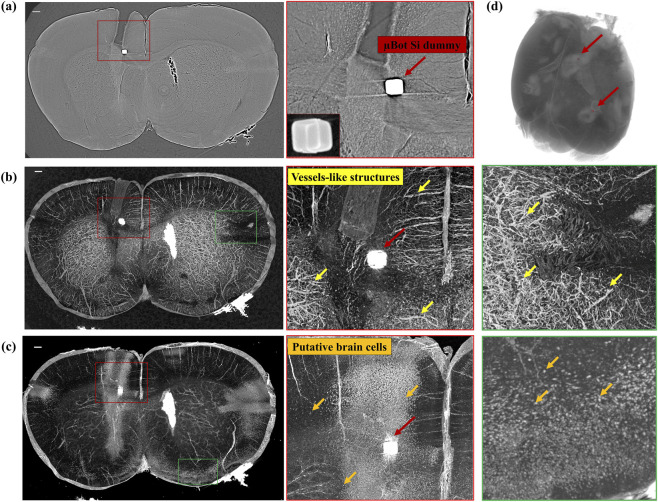
XPCT segmented images. **(a)** Reconstructed XPCT image of a 30-slice-thick brain slab, acquired with a δ/β ratio of approximately 10, highlighting the presence of the μBot dummy (red arrow) embedded within the brain tissue. The bottom-left panel shows a zoomed-in 3D rendering of the μBot dummy. **(b)** Minimum intensity projection (MIN) with inverted contrast, emphasizing vascular-like structures, visible as bright signals (yellow arrows indicate representative vessels). **(c)** Maximum intensity projection (MAX) with non-inverted contrast, enhancing the visibility of putative brain cells, shown as bright signals (orange arrows indicate examples). **(d)** 3D rendering of the whole brain volume, with red arrows indicating the two µBot Si dummies implanted in the right hemisphere. Scale bar: 100 µm.

To overcome the limitations encountered with 2D histology and X-CLARITY, we next explored advanced three-dimensional imaging approaches. Among these, XPCT is well established in the literature for its ability to provide high-resolution, label-free visualization of soft tissues, making it particularly suitable for investigating brain microarchitecture. X-ray phase contrast images acquired in propagation mode were reconstructed to highlight the dense dummies within the surrounding soft tissues (Figure 2a). To further enhance the contrast and enable 3D interpretation, we exploited the z-projection of maximum and minimum intensities, which consists in projecting on the visualization plane the voxels of a set of continuous slices. Each pixel of the output image contains the maximum or the minimum gray level found along the axis perpendicular to that pixel. We report in Figure 2b the segmentation of the vasculature, with vessels-like structures emphasized in white, while Figure 2c highlights high-density structures indicating putative brain cells. These high-density structures and vascular features were visualized in XPCT images based on previously established morphological criteria and contrast characteristics (Cedola et al., 2017; Massimi et al., 2019; Maugeri et al., 2023). XPCT also enables 3D-rendering of the whole acquired brain volume in different imaging modalities (Supplementary Figures S4a-c) in which the dummies can be clearly identified at their implantation sites (Figure 2d). Notably, the full geometry of the dummies is preserved during images’ reconstruction, as highlighted in the detailed panel in Figure 2a. Moreover, XPCT allows for virtual slicing of the sample in any desired plane or direction prior to subsequent standard histological methods (Supplementary Figures S4d,e), thereby facilitating a comprehensive spatial framework for assessing spatial apposition of the dummies in all orientations. Across all XPCT acquisitions, all dummies implanted in the two brains analyzed were successfully localized (5/5, 100%), with no displacement or loss observed, confirming the reliability of this approach for non-destructive 3D localization of microscale implants, as reported in Table 2.

These results validate XPCT as a promising *ex vivo* technique for detailed visualization of both the dummies and the surrounding brain tissue, which were not simultaneously accessible with the other techniques evaluated here. The vascular-like structure and putative brain cells visualized through XPCT will be further validated in the future *in vivo* studies, using targeted histological markers, enabling a direct correlation between 3D imaging findings and molecular-level tissue characterization to assess tissue-device structural interface.

### Micro-CT imaging for spatial localization of µBot Si dummies

Given the limited accessibility, high cost, and time constraints associated with XPCT, conventional micro-CT was subsequently evaluated as a practical alternative.

**FIGURE 3:**
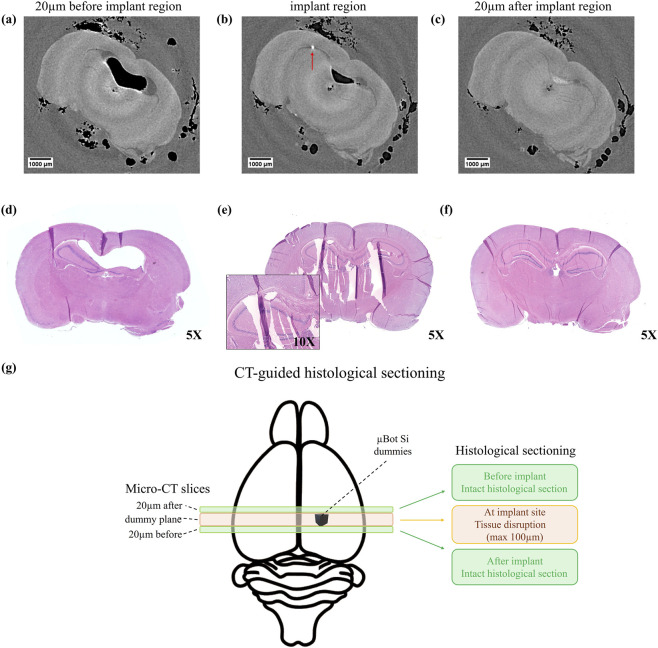
CT-guided histological sectioning for precise localization of µBot Si dummies. **(a–c)** Consecutive micro-CT slices of the brain region containing the µBot Si dummy implanted in the left hemisphere. **(a)** Section 20 µm before the implant, **(b)** slice at which the dummy first becomes visible (indicated by a red arrow), **(c)** slice located 20 µm beyond the implant. Scale bar: 1000 µm. **(d–f)** H&E-stained histological sections collected around the implant site using the CT-guided serial sectioning strategy. **(d)** Morphologically intact section immediately before the implant region, **(e)** section at the implant region, showing tissue disruption caused by dummy interaction with the microtome blade, **(f)** morphologically intact section immediately after the implant region. Objectives used for tile scan acquisition are indicated in the panels. **(g)** Schematic overview of the CT-guided sectioning workflow illustrating the sampling strategy relative to the predicted implant depth.

Figures 3a–c show consecutive micro-CT slices of a brain region containing a µBot Si dummy. Specifically, Figure 3a corresponds to a slice located 20 µm prior to the implant, Figure 3b shows the slice in which the dummy is visible, and Figure 3c represents a slice 20 µm beyond the implant. This approach enabled precise localization of the dummy within the entire reconstructed brain volume. However, due to the limited spatial resolution of micro-CT, no cellular or tissue details could be resolved, thus requiring additional histological analysis. To address the limitations of conventional histology highlighted in Figure 1, micro-CT data were used as a spatial guide for targeted histological sectioning. The depth of the dummy was determined by measuring the distance between the first CT slice in which brain tissue appeared and the first slice in which the dummy became visible (distance = 3.02 mm). Based on the 20 µm spacing between CT slices, histological sectioning was performed by collecting five consecutive sections (4 µm thickness each), discarding 40 µm of tissue between sampling points. This strategy enabled controlled serial sectioning in proximity to the implant, effectively “slowing down” the microtomy process compared to standard protocols. Starting approximately 200 µm before the predicted dummy location, all sections were collected to preserve tissue continuity. Although the interaction between the rigid dummy and the microtome blade induces localized tissue damage, the extent of this disruption is confined to a maximum of 100 µm—corresponding approximately to the size of the dummy—around the implant region. This approach allows the recovery of morphologically intact sections immediately adjacent to the implant site, both preceding and following the dummy region (see schematic overview in Figure 3g).

In detail, the histological section corresponding to the implant region (Figure 3e) still exhibits substantial disruption, whereas the sections collected immediately before (Figure 3d) and after the implant (Figure 3f) are morphologically intact, enabling reliable evaluation of the tissue surrounding the implantation site.

A second µBot Si dummy was implanted in the contralateral (right) hemisphere of the same animal, as reported in Table 1. This implant was localized at a depth of 2.60 mm, as determined by the same micro-CT-guided sectioning approach described above. The same serial sectioning strategy was applied, and the resulting histological sections are presented in Supplementary Figure S5. The outcome corresponds to approximately 25 sections lost per dummy (∼100 µm ÷ 4 µm section thickness), as reported in Table 2.

Overall, while micro-CT alone is insufficient for resolving cellular features, it provides a powerful complementary tool for accurate implant localization and for guiding histological sectioning. Compared to conventional histology, which results in loss of spatial information and tissue integrity (Figure 1), this combined approach preserves the tissue surrounding the implant, despite the unavoidable loss of the ∼100 µm region occupied by the dummy. Importantly, it enables consistent retrieval of intact sections immediately adjacent to the implantation site at micrometer-scale resolution. This level of spatial control is critical for future analyses of the local tissue response, including the foreign body reaction (FBR), in a reliable and anatomically meaningful manner.

Taken together, as shown in Table 3, each modality presents distinct and complementary limitations. Classical histological sectioning is inherently destructive in the region of implant presence, as the rigid dummy damages the microtome blade, leading to irreversible loss of sections precisely where the device resides. Moreover, standard microscopy is restricted to two-dimensional evaluation of thin sections. CLARITY-based approaches, while enabling volumetric imaging, are associated with substantial tissue deformation, including swelling and structural distortion (ranging from approximately 5%–30% depending on the protocol). Micro-CT, although more widely accessible and capable of non-destructive volumetric imaging for accurate dummy localization, provides insufficient soft tissue contrast to resolve brain cytoarchitecture. XPCT, on the other hand, offers high-resolution, high-contrast three-dimensional imaging with sub-micrometric detail and preserves morphological integrity, enabling precise visualization of putative-cellular and vascular-like structures in close proximity to the implants; however, its application remains constrained by the limited accessibility of synchrotron beamlines. Overall, no single modality fulfills all the requirements for a comprehensive analysis of µBot Si dummies in brain tissue, highlighting the need for a multimodal approach as the one reported in the present Brief Research Report.

**TABLE 3:** Comparison of standalone imaging and histological modalities for µBot Si dummy analysis in brain tissue: individual capabilities and limitations.

Characteristics	Histological sectioning	Micro-CT	XPCT
Sample preparation	Paraffin embedding after post-fixation	Paraffin embedding after post-fixation	Paraffin embedding after post-fixation
Destructive vs. non-destructive	Destructive: The dummy damages the microtome blade, causing loss and tearing of sections in the region of implant presence; material in the vicinity of the device is irreversibly lost	Non-Destructive: The sample remains intact and fully available for subsequent analyses	Non-Destructive: The sample remains intact and compatible with downstream histological processing
Voxel/Resolution scale	Micrometric resolution in the section plane (1–5 µm section thickness); inter-section spacing variable (5–100 µm)	Isotropic voxel size of 20 µm in the present study	Isotropic voxel size of 3 µm in the present study, enabling sub-cellular structural details
Implant detectability	Not possible as standalone: The dummy destroys the microtome blade and sections are lost precisely in the region of interest	100%: The implant is unambiguously localized in 3D, enabling image-guided sectioning	100%: The implant is precisely localized in 3D with high spatial accuracy
Soft tissue contrast	Excellent: Selective staining (e.g., H&E, immunofluorescence) provides cellular and sub-cellular discrimination	Low: Brain tissue shows poor intrinsic X-ray attenuation contrast; white matter, grey matter, and surrounding structures are difficult to distinguish without contrast enhancement	Good to excellent: Phase-contrast retrieval provides intrinsic contrast on soft tissues without staining, enabling differentiation of brain tissue compartments
Suitability for downstream histology	N/A: this is itself the reference standard; serial sections allow additional staining	Fully compatible: Sample unaltered; histological processing can follow scanning without restrictions	Fully Compatible: Sample unaltered; histological sectioning can be performed after scanning
Major artifact/Failure mode	Section tearing and material loss in the dummy region; variable inter-section gap; operator-dependent cutting quality	Ring artifacts from rotation-based acquisition; partial volume effect at tissue interfaces; limited spatial resolution	Phase retrieval artifacts (halos, ringing at sharp interfaces); sensitivity to mechanical vibrations; motion artifacts during acquisition
Accessibility/Cost	High accessibility: Standard equipment in pathology and research laboratories; low to moderate per-sample cost	High accessibility: Micro-CT systems widely available in research institutions; moderate cost per acquisition	LOW accessibility: Requires synchrotron beamline access (e.g., ESRF, elettra, diamond); high cost; competitive peer-reviewed allocation required

Abbreviations: XPCT, X-ray Phase-Contrast Tomography; micro-CT, micro-computed tomography; H&E, hematoxylin and eosin. N/A, not applicable; Resolution values refer to the acquisition parameters used in the present study.

These limitations are reflected in the quantitative outcomes summarized in Table 2: even though micro-CT and XPCT achieved complete dummy localization (5/5, 100%), only the combination of 3D imaging with targeted histological sectioning enables simultaneous spatial localization of the implant and high-resolution characterization of the surrounding tissue. These complementary strengths informed the design of the integrated workflow described below.

### Final workflow for µBot Si dummies localization

**FIGURE 4:**
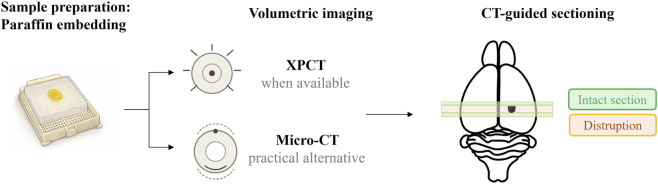
Final workflow for µBot Si dummies localization. Sample preparation (paraffin) is followed by volumetric imaging via two alternative approaches: XPCT, when available, or micro-CT as a practical alternative. Both methods provide spatial guidance for CT-guided histological sectioning, enabling controlled serial sectioning in proximity to the implant. The resulting histological sections immediately before and after the implant site are morphologically intact (green), while sections spanning the implant region show tissue disruption caused by interaction with the microtome blade (orange, max 100 µm).

Building on the complementary strengths of each modality, the final workflow for analyzing µBot Si dummies combines 3D imaging and histological sectioning (Figure 4). Sample preparation is followed by volumetric imaging: when available, XPCT provides detailed visualization of tissue surrounding the implanted dummy. This technique is also suitable to perform a spatial analysis of the surrounding soft tissue compartments—primarily distinguishing putative brain cellular and vascular-like components—in relation to the implanted devices. Micro-CT represents a practical and readily accessible alternative for dummy localization and for guiding subsequent histological analysis. In fact, despite the different spatial resolutions (a few micrometers vs. 20 micrometers), the resolution is sufficiently high in both cases to support the same workflow, and the imaging-guided sectioning approach can therefore be applied with both 3D imaging methods. This integrated approach preserves tissue adjacent to the implant and overcomes the limitations of stand-alone histology.

## Discussion

Neurostimulation has the potential to treat neurodegenerative diseases and psychiatric disorders by modulating brain activity and alleviating symptoms associated with these conditions. Current neuromodulation techniques, such as DBS, face limitations due to low spatial and temporal resolution, as well as invasiveness. The CROSSBRAIN project seeks to address these challenges by utilizing wireless microbots (µBots) of micrometer size for precise and minimally invasive brain modulation. However, the potential MRI-incompatibility of these microdevices poses an additional challenge for their monitoring and evaluation. Non-destructive, high-resolution imaging methods are therefore essential for assessing micro-scale implants, particularly when achieving the required spatial resolution (i.e., sub-50 µm voxels) with MRI would be impractical without employing ultra-high-field systems (>11T) (Marques and Norris, 2018; Tohka, 2014; Boulant et al., 2024; Mulder et al., 2019; Dieringer et al., 2013; Winter et al., 2021). Advanced imaging techniques are thus essential for optimizing implant placement and will represent a critical tool for the future *in vivo* assessment of FBR once functional devices are implanted.

### Demonstrated findings: 3D imaging combined with histology as practical workflow

In this study, we established and validated an *ex vivo* integrated workflow for accurately visualizing µBot Si dummies within fixed mouse brain tissue. Our results first demonstrate that traditional 2D imaging techniques, such as standard microscopy with H&E staining, have limited efficacy in assessing the positioning and tissue structural interface, *i.e.*, the spatial distribution of cells and vessels around the µBot Si dummies. The main challenge lies in preserving the position of the dummies during the tissue sectioning and staining process. Its displacement, particularly after staining, and the disruption of the surrounding tissue during sectioning hinder the ability to effectively evaluate dummy localization.

The X-CLARITY protocol highlights key limitations associated with tissue-clearing-induced morphometric changes. Although this method enables three-dimensional imaging of intact tissue, it did not allow precise tracking of the position of the µBot Si dummies. In particular, tissue swelling occurring during the clearing process, combined with minor displacement of the implants, further complicated accurate localization. Such distortions are not specific to X-CLARITY but represent a general limitation of clearing approaches: hydrophilic methods typically induce tissue expansion on the order of a few percent (∼5–10%), whereas solvent-based protocols more commonly result in shrinkage and deformation (∼10–30%, depending on conditions) (Ertürk et al., 2012; Chung et al., 2013). Importantly, although the direction of deformation differs between these two classes of protocols, in both cases the magnitude of macroscopic morphological changes is substantial and well above the spatial scale of the micro/nanostructures investigated here.

A critical compounding factor in the present *ex vivo* setting is that the µBot dummy structures were not mechanically coupled to the surrounding tissue—a condition that, under *in vivo* implantation, would likely be mitigated by progressive tissue integration and encapsulation of the device. However, even assuming full mechanical coupling *in vivo* between implant and tissue, a potential source of spatial inaccuracy would likely persist: the µBot structures are rigid and dimensionally invariant and may therefore not deform in concert with the surrounding tissue during the clearing process. As the tissue undergoes volumetric changes driven by the clearing chemistry, the implant is expected to remain largely unaffected, potentially introducing a relative spatial mismatch between device and tissue. This effect is particularly consequential given the micrometric dimensions of the µBot Si dummy structures (∼100 µm) under investigation in the present study: even a conservative 5% tissue expansion corresponds to absolute spatial displacements of several micrometers in the peri-implant region, a magnitude comparable to the size of individual cells and subcellular structures relevant to the tissue response for *in vivo* future investigation.

While X-CLARITY remains valuable for volumetric visualization of biological structures, our findings indicate that caution is required when using clearing-based approaches to assess implant positioning and tissue–device structural interface. Complementary imaging strategies that preserve or independently reconstruct spatial references are therefore necessary for reliable microscale localization.

A critical factor underlying the performance of any 3D imaging approach in this context is the relationship between imaging resolution, voxel size, and µBot detectability. When the voxel size approaches or exceeds the characteristic dimensions of the µBots, partial-volume effects become substantial. Partial-volume phenomena are well documented in volumetric imaging literature and arise when multiple materials or tissue classes are contained within the same voxel, leading to signal averaging and reduced boundary definition (Tohka, 2014; González Ballester et al., 2002). In practice, imaging and segmentation studies generally indicate that dense structures within brain tissue should span a few voxels (≈2 or more) (Buzug, 2008; Kak and Slaney, 2001) across their diameter to maintain adequate contrast and geometric fidelity for reliable detection. Since the µBots Si dummies investigated in this study have dimensions on the order of hundred micrometers, voxel sizes approaching this scale result in the device signal being averaged with the surrounding tissue, thereby reducing contrast and making structures occupying only one or two voxels difficult to clearly distinguish from the background. In this context, isotropic imaging modalities such as micro-CT and XPCT are well suited for resolving the µBot dimensions studied here. Conventional laboratory micro-CT typically achieves voxel sizes in the range of tens of micrometers (≈10–50 µm depending on system configuration and sample-detector geometry (Kak and Slaney, 2001), whereas XPCT can reach isotropic spatial resolutions on the order of a few micrometers (≈1–5 µm) in propagation-based implementations (Wilkins et al., 1996).

Consistent with these considerations, our results demonstrate that micro-CT provides a reliable solution for spatially localizing the Si dummies within the brain, enabling virtual slicing of the brain tissue and precise identification of the dummy’s position. However, due to its limited soft tissue contrast, micro-CT alone is insufficient for directly assessing the interaction between the dummy and the surrounding tissue, thus requiring integration with histological analysis. XPCT, when available, overcomes this limitation by offering high-contrast volumetric imaging a priori, enabling detailed visualization of cellular and vascular structures without the need for tissue sectioning, which is particularly valuable when cutting could compromise sample integrity. However, when a more specific cellular discrimination is required, such as distinguishing between neurons and astrocytes, conventional histological approaches remain necessary, as they allow for targeted immunolabeling and *post hoc* identification of distinct cell populations (Maugeri et al., 2023; Donato et al., 2024).

Micro-CT and XPCT therefore play complementary roles: the former provides a practical and widely accessible method to guide targeted histological sectioning, ensuring preservation of tissue adjacent to the implant, while the latter emerged as the most promising technique for simultaneously localizing the µBot Si dummies and analyzing spatial apposition at the implantation site, offering the best compromise between implant detectability and soft tissue contrast among all methods tested. Together, these approaches support the design of an integrated workflow combining 3D imaging with standard histological sectioning for spatially precise studies and validation of tissue-device structural interaction.

### Future directions

Thanks to the methodology presented in this Brief Research Report, future studies will enable a comprehensive assessment—extending up to several months post-implantation—of the FBR of brain tissue to µBot implantation within the CROSSBRAIN framework. The *in vivo* implantation methodology of functional CROSSBRAIN µBots and the resulting tissue response will be the focus of future investigations. These investigations will directly benefit from the multimodal workflow and experimental pipeline established in this study, which enables the precise collection and registration of tissue sections in relation to each individual µBot. This integrated approach provides a robust framework for systematically assessing local tissue responses at the device–tissue interface, including neuroinflammatory processes and cell death, in proximity to each implanted µBot.

Among the modalities evaluated, XPCT emerges as uniquely capable of providing high-resolution volumetric visualization of the brain microenvironment in close proximity to the implanted devices, enabling assessment of the spatial distribution of soft tissue compartments—most notably putative cellular and vascular-like structures—at a level of detail not achievable with conventional micro-CT or standard histological approaches alone. This capability is of direct relevance to the CROSSBRAIN project: understanding how cellular density and vascular organization are spatially arranged around each device is essential for designing and optimizing future neuromodulation strategies. XPCT therefore provides a unique and currently indispensable imaging tool for bridging device localization with microenvironmental tissue characterization in complex brain implantation scenarios.

Importantly, as these µBots are ultimately envisioned as functional systems capable of interacting with brain tissue through both neural recording and stimulation, detailed knowledge of the spatial distribution of surrounding cellular components becomes crucial. Such information is essential to correlate device performance—particularly in terms of recording fidelity and stimulation efficacy—with the quality and nature of their embedding within the target neural tissue, which defines the effective interface with the excitable brain microenvironment.

Thanks to the multimodal strategy developed in this study, it will be possible not only to perform detailed histological investigations of the tissue and assess FBR in the presence of the functional implanted device, but also, through integration with XPCT datasets, to map and correlate the spatial distribution of different cellular populations, including neurons and glial cells. This combined approach will enable a deeper investigation of the biological and functional mechanisms induced by the µBots within brain tissue, as a function of the specific cellular environments with which they interact.

## Conclusion

Neurostimulation techniques are increasingly being developed to treat a range of brain disorders, with a clear trend toward the miniaturization of implantable devices to reduce their physical footprint and overall impact on the surrounding tissue. While this miniaturization can help attenuate the FBR, it also makes the devices progressively more difficult to visualize within brain tissue, particularly when they are not MRI-compatible. This Brief Research Report presents a method for acquiring detailed information on the neuro-device tissue interface. Although it was developed within the framework of the European CROSSBRAIN project, which involves the use of ∼100 µm-scale microdevices, the approach is not limited to this specific application and can be extended in the future to collect comparable information from a wide range of other implantable devices.

Our findings highlight the challenges in monitoring small implants and their interaction with neural tissue, revealing significant differences in imaging effectiveness. While standard microscopy and X-CLARITY were limited by tissue disruption and device displacement, CT imaging effectively localized µBots and guided histological sectioning. Notably, XPCT provided detailed three-dimensional visualization of µBot Si dummies positioning and structural interface between tissue and device, offering valuable insights for future evaluation of FBR and the integration of new neuromodulation devices. Importantly, these experiments were conducted *ex vivo* to establish and validate a workflow for post-implantation visualization. Extending this approach to future *in vivo* studies and at chronic time points will be fundamental to fully assess implantation reliability, tissue responses, and the translational potential of next-generation, minimally invasive and MRI-incompatible devices used for brain stimulation. Even if the implants will be positioned with precise stereotactic coordinates, post-implant histological identification of these microscale devices would remain challenging, highlighting the essential role of 3D imaging techniques.

## Data Availability

The original contributions presented in the study are included in the article/Supplementary Material, further inquiries can be directed to the corresponding author.
