# Supplementary material for: Ex vivo localization of wireless implantable microdevice using high-resolution 3D imaging techniques
Source: Front Bioeng Biotechnol. 2026 Jun 2;14:1830115. doi: 10.3389/fbioe.2026.1830115 (PMC13269262; doi:10.3389/fbioe.2026.1830115)
Supplement: Supplementary file 1 [file DataSheet1.pdf]

Ex vivo localization of wireless implantable microdevice using high-resolution 3D imaging techniques

Supplementary Materials

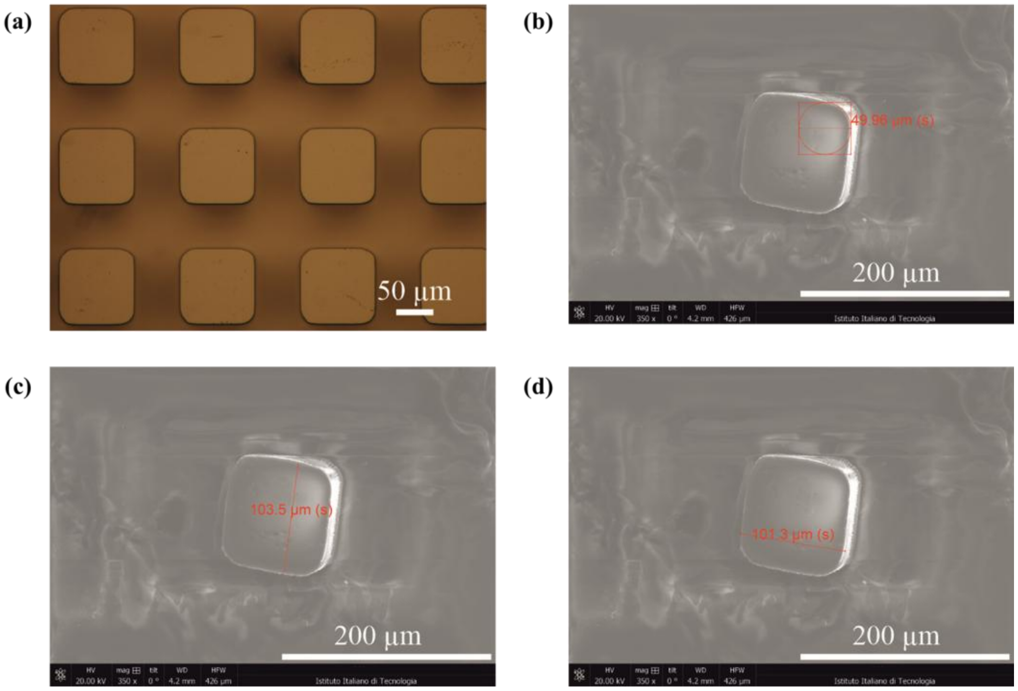

Figure S1. Pictures of the µBot Si dummies. (a) Microscopic image after the silicon dry-etching. (b), (c) and (d) Scanning Electron Microscopy (SEM) image indicating the relevant dimensions, the surface morphology, and structural features. Scale bars are included for reference.

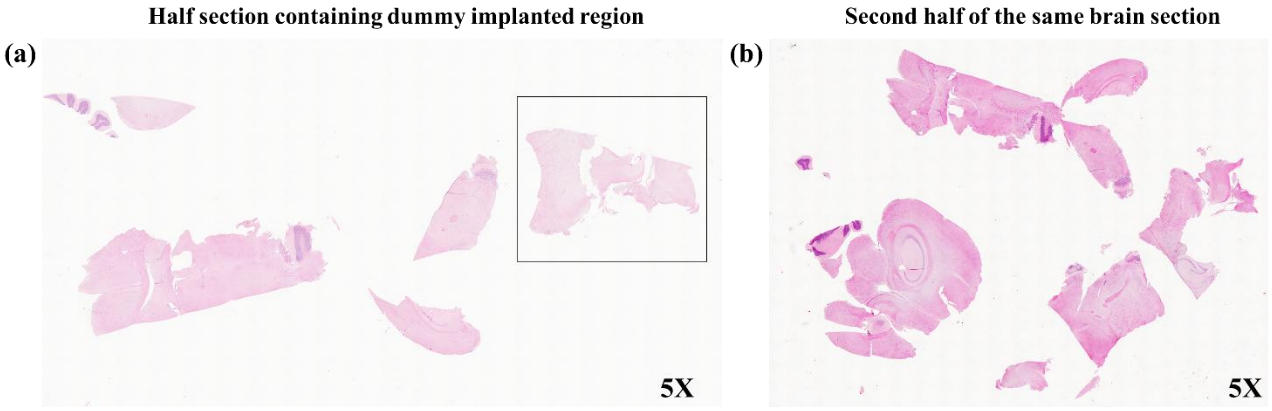

Figure S2. ***Histological sections at the implant site following mechanical disruption induced by the dummy.*** (a) Full view of the first tissue half; the region surrounding the implant site is highlighted by a black box. (b) The corresponding second half of the same section. Together, (a) and (b) represent the fragments resulting from the splitting of a single tissue section at the implant site during histological processing. Images acquired as tiled scans at 5× objective magnification. Sections are stained with H&E.

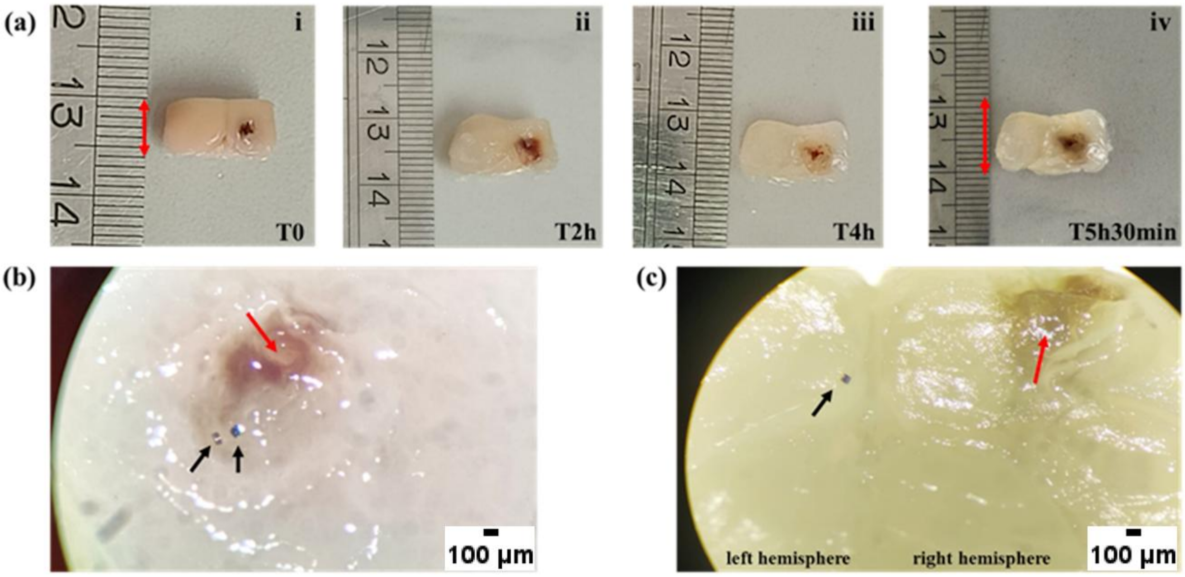

Figure S3. Images of implanted brain tissue following the X-CLARITY clearing protocol. (a) Swelling of the brain tissue observed during the clearing process. Red double-headed arrows indicate the expansion of the brain size, increasing from 0.5 cm at time point (i) to 1 cm at time point (iv). The specific time points corresponding to the clearing stages are labeled in the images. (b) Black arrows indicate two µBot Si dummies that were displaced from the implantation site, which is marked by a red arrow. (c) The red arrow highlights the original implantation area in the right hemisphere, while the black arrow points to a third dummy that migrated into the contralateral hemisphere (left) during the clearing protocol. Scale bars are included as reference.

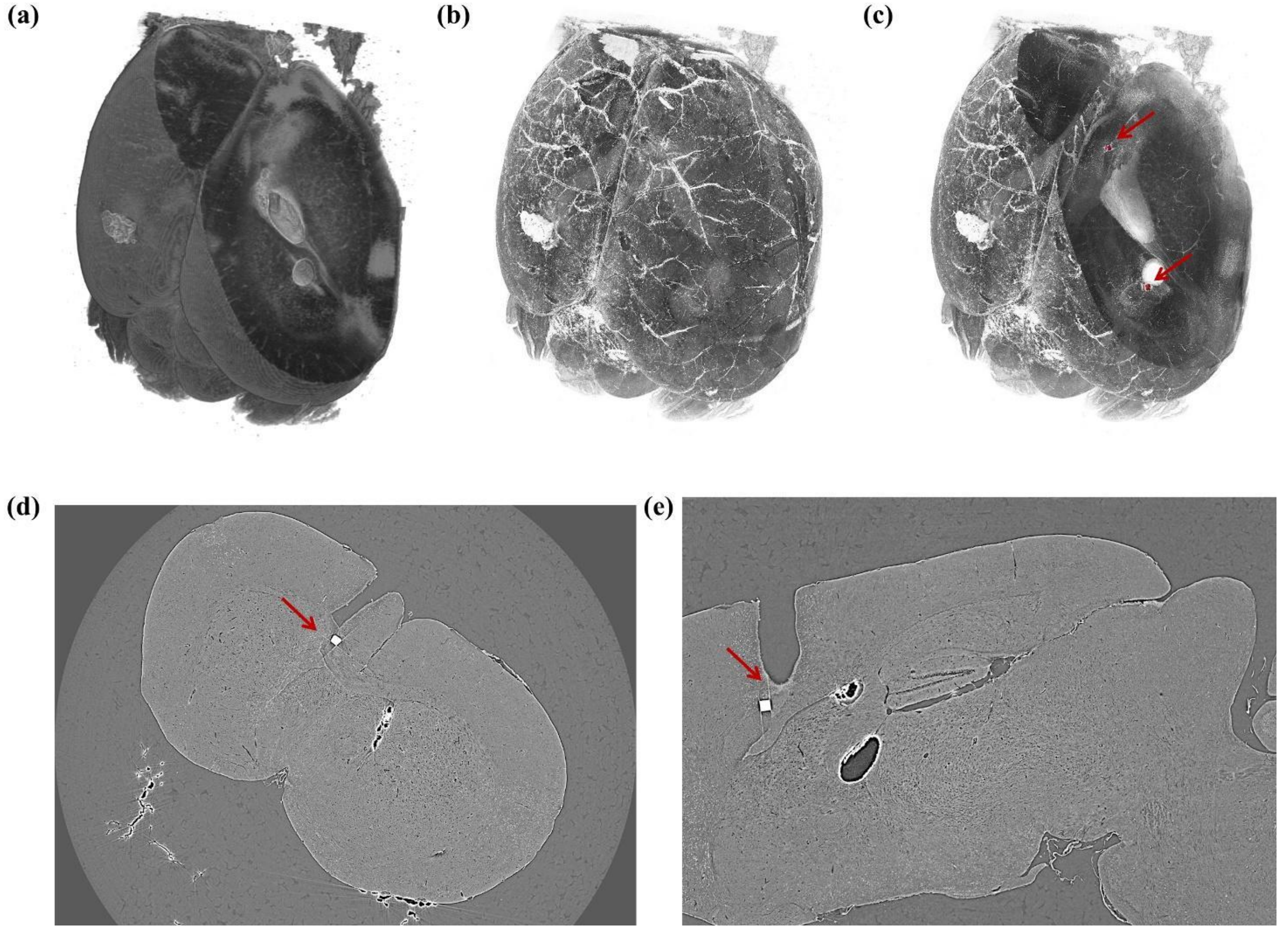

Figure S4. Representative images of XPCT slicing virtual modality. 3D-rendering of the different imaging modalities (a) absorption reconstruction. (b-c) Minimum intensity projection (MIN) with not-inverted contrast emphasizes vascular structures; red arrows point the implanted μbot Si dummies. Red arrows point the implanted μBot Si dummies in the same brain region observed using coronal view (d) and sagittal view (e) of the absorption image modality reconstruction.

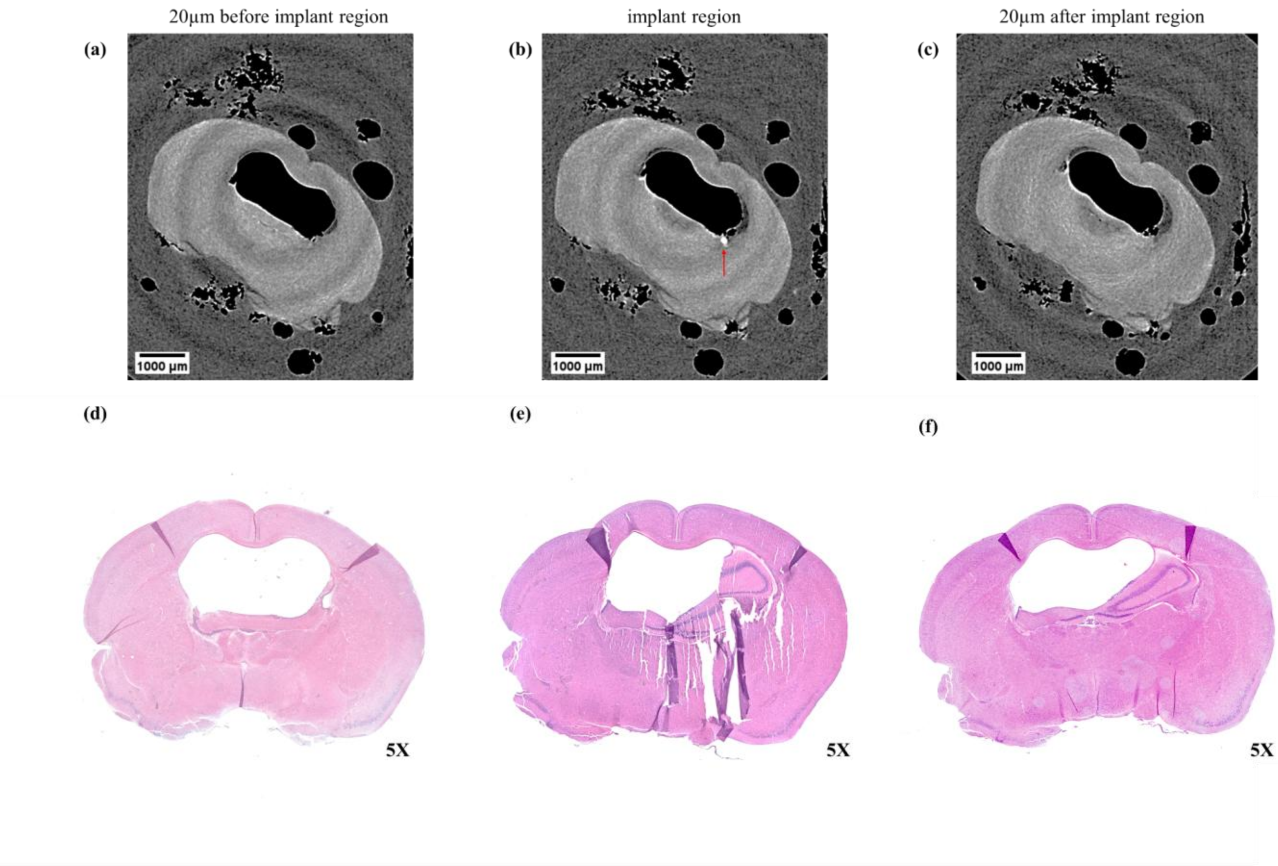

Figure S5. ***CT-guided histological sectioning of the µbot Si dummy implanted in the right hemisphere.*** (a–c) Consecutive micro-CT slices of the brain region containing the dummy. (a) Slice located 20 µm before the implant, (b) slice at which the dummy first becomes visible (indicated by a red arrow), (c) slice located 20 µm beyond the implant. Scale bar: 1000 µm. (d–f) H&E-stained histological sections collected around the implant site using the CT-guided serial sectioning strategy (see Figure 3 for details). (d) Morphologically intact section immediately before the implant region, (e) section at the implant region, showing tissue disruption caused by interaction with the microtome blade, (f) morphologically intact section immediately after the implant region. Objectives used for tile scan acquisition are indicated in the panels.
